# Detection and Characterization of Damage in Quasi-Static Loaded Composite Structures Using Passive Thermography

**DOI:** 10.3390/s18103562

**Published:** 2018-10-20

**Authors:** Joseph Zalameda, William Winfree

**Affiliations:** NASA Langley Research Center, MS231 Hampton, VA 23681, USA; william.p.winfree@nasa.gov

**Keywords:** nondestructive evaluation, passive thermography, composite hat stiffened panel, quasi-static seven-point load, two-dimensional quadrupole thermal model, matrix crack, delamination damage depth, fracture heating

## Abstract

Real-time nondestructive evaluation is critical during composites load testing. Of particular importance is the real time measurement of damage onset, growth, and ultimate failure. When newly formed damage is detected, the loading is stopped for further detailed characterization using ultrasound inspections or X-ray computed tomography. This detailed inspection data are used to document failure modes and ultimately validate damage prediction models. Passive thermography is used to monitor heating from damage formation in a hat-stiffened woven graphite epoxy composite panel during quasi-static seven-point load testing. Data processing techniques are presented that enable detection of the small transient thermographic signals resulting from damage formation in real time. It has been observed that the temperature rise due to damage formation at the surface is composed of two thermal responses. The first response is instantaneous and conforms to the shape of the damage. This heating is most likely due to irreversible thermoelastic, plastic deformation, and microstructural heating. The second response is a transient increase in temperature due to mechanical heating at the interface of failure. Two-dimensional multi-layered thermal simulations based on quadrupole method are used to investigate the thermal responses. In particular, the instantaneous response is used as the transient response start time to determine damage depth. The passive thermography measurement results are compared to ultrasonic measurements for validation.

## 1. Introduction

The development of progressive damage analysis (PDA) models allows for predicting damage in composite structures and thus determines the residual strength and ultimately the remaining life. PDA models can potentially help to eliminate testing requirements and thus decrease certification times for new composite materials and designs [1]. In addition, PDA models can be used as a tool to assess the safety of as built structures which might have manufacturing imperfections or service damage. PDA models, however, require validation [2]. Nondestructive evaluation is used as a tool to validate PDA models to enable comparisons between measured damage vs. predicted damage [1,2]. Work being performed at NASA Langley involves the development of PDA models [3,4,5,6,7,8] and building an nondestructive evaluation (NDE) database of measured damage under both fatigue and quasi-static testing of composite structures. The challenge is to incrementally control the grow of the damage during loading and document the damage growth using ultrasound or X-ray CT (computed tomography). To incrementally grow the damage, real-time NDE is required. Real-time NDE allows for monitoring and controlling the growth of the damage as a function of the applied load. In this work, a quasi-static bending load is applied to a single stringer stiffened composite panel. When damage is detected, the loading is stopped and another inspection technique such as ultrasound or X-ray CT provides a detailed assessment of the panel damage as a function of depth. When the structure is loaded to failure, real-time NDE records the damage leading up to failure. The purpose of this paper is to describe the detection of damage onset and characterize the damage depth during quasi-static loading using passive thermography.

Passive thermography is similar to active thermography where both techniques rely on detecting small surface temperature differences due to applied or removed energy. Active thermography implies the control of the energy, such as start time, duration, amount of energy delivered, and frequency for the purposes of inspection. Examples of active thermography heat sources are flash lamp, quartz lamp, eddy current, microwave, and ultrasound [9,10,11,12,13]. Passive thermography implies no control of the applied energy such as the sun, hot air, moisture evaporation, and structural loading. The infrared (IR) camera passively records the thermal imagery. A sample of the numerous studies performed use passive thermography for damage detection during cyclic fatigue structural loading is given here [14,15,16,17,18,19]. The challenge for passive thermography during quasi-static loading is to detect the generated nonrepetitive, small, transient thermal signals. Previous works using passive thermography during static loading on metals [20], glass fiber reinforced composites [21,22,23], and on carbon fiber composites [24,25,26,27] have been performed. These efforts mainly focused on coupon level testing. In this work, single stringer hat-stiffened panels were subjected to quasi-static loading. The presented data processing techniques enable detection of the small transient thermographic signals resulting from damage formation. In addition, two-dimensional multi-layered thermal simulations based on quadrupole method are used to investigate the thermal responses as a function of depth for quasi-static loading. The passive thermography measurement results are validated with ultrasonic measurements.

## 2. Material and Method

### 2.1. Sample Description

**Figure 1:**
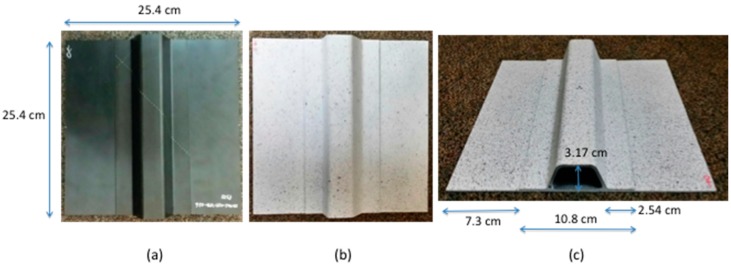
Single stringer hat-stiffened composite panel: (**a**) stringer side; (**b**) stringer side painted for digital image correlation measurements; (**c**) cross sectional side view.

The hat-stiffened single stringer composite panel skin was 12 plies with a thickness of 0.22 cm; the stiffener flange was 12 plies thick with a thickness of 0.24 cm. The skin material was an IM-7 tape with 8852 matrix and the stiffener was a woven composite AS4 Fabric with 8852 matrix. The dimensions of the panel are shown in Figure 1. Figure 1a–c show the stiffened composite panel stringer side, a painted specimen, and a cross sectional view, respectively. The panel was painted for digital image correlation measurements to record panel out-of-plane deformation. The paint did not affect the passive thermography measurements. Digital image correlation results are not discussed in this paper.

### 2.2. Method for Loading

**Figure 2:**
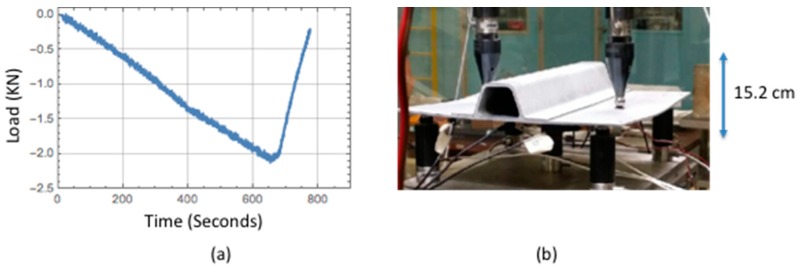
Single stringer hat-stiffened composite panel applied quasi-static load: (**a**) example applied load as a function of time; (**b**) panel deformation due to seven-point bend test.

Quasi-static loads were applied using seven application points: two on top (located in middle just outside of the flange) and five on the bottom (located at each corner and center). The load was applied from the bottom while the top was held stationary at two points. This seven-point bend test configuration allowed for panel deformation that results in damage formation between the stiffener flange and skin. The load was applied collectively from the bottom five pins and is measured with a load cell attached to the two top pins. Example of a typical applied load for a given test panel and deflection are shown in Figure 2a,b respectively. The load rate was applied under displacement control and was reduced past a load point where damage formation might occur. For a typical load run as shown in Figure 2a, the load rate was reduced from −0.0033 Kilonewtons/s to −0.0020 Kilonewtons/s. The applied quasi-static maximum load varied from 2.22 to 4.45 Kilonewton, and for each load run, the maximum applied load would increase successively as new damage was formed. After each load run the panel was removed for inspection. Twelve panels were tested and load runs for each panel varied up to a maximum of fifteen runs.

### 2.3. Passive Thermography Measurement System

**Figure 3:**
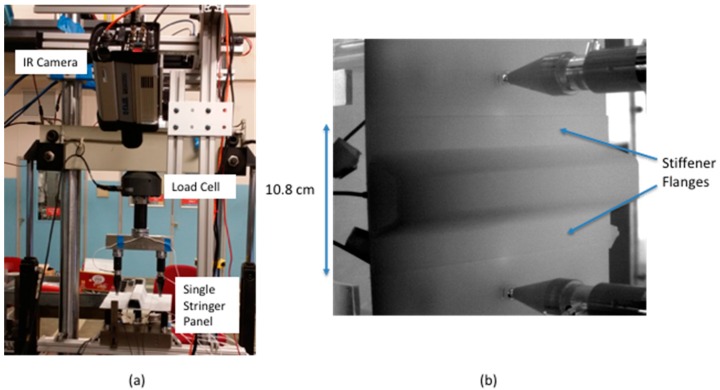
Passive thermography test setup (**a**) with IR camera view (**b**).

Passive thermography was used to track damage on the stringer side. The passive inspection captured the thermal variations in real time during the quasi-static loading. When damage was detected, the loading was stopped and the sample is removed for further damage characterization using ultrasound or X-ray CT [28]. The test setup is shown in Figure 3a along with the IR camera view Figure 3b. The basic system consists of a cooled IR camera and an image data acquisition computer. The IR camera specifications are listed in Table 1. The setup required a Plexiglas^®^ shield to filter out spurious IR background sources (not shown in Figure 3a). The camera frame rate was externally triggered. The load signal was also acquired using a USB based 12-bit data acquisition module. For each IR camera frame, a load value was acquired. A typical test would last 15 to 20 min, and up to 60 gigabytes of thermal data would be acquired for each run. The IR camera was connected to a custom developed data acquisition system (via GigE interface) for image acquisition. The custom developed image acquisition system allowed for simultaneous acquisition of load data, real-time processing of the IR imagery using averaging, delayed subtraction, and real time contrast adjustment.

### 2.4. Detection of Damage

**Figure 4:**
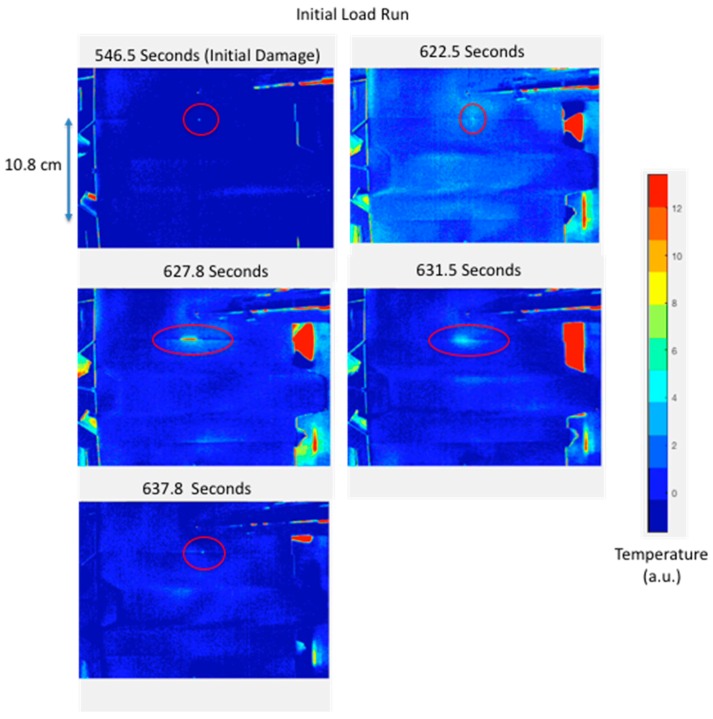
Processed thermal inspection images during initial quasi-static load run.

**Figure 5:**
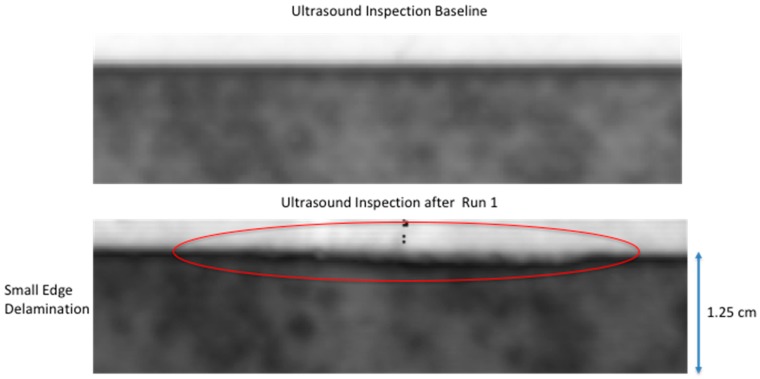
Ultrasonic inspection image after initial quasi-static load run with comparison to baseline ultrasonic inspection.

**Figure 6:**
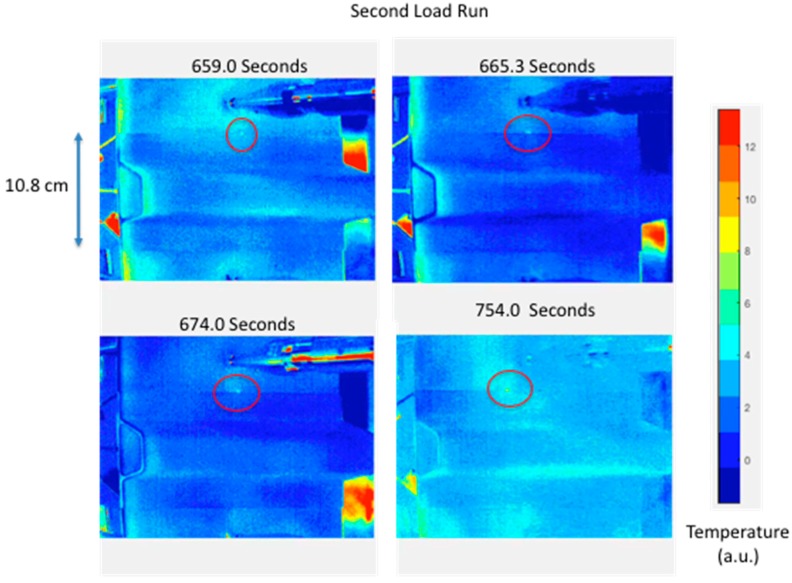
Processed thermal inspection images during second quasi-static load run.

**Figure 7:**
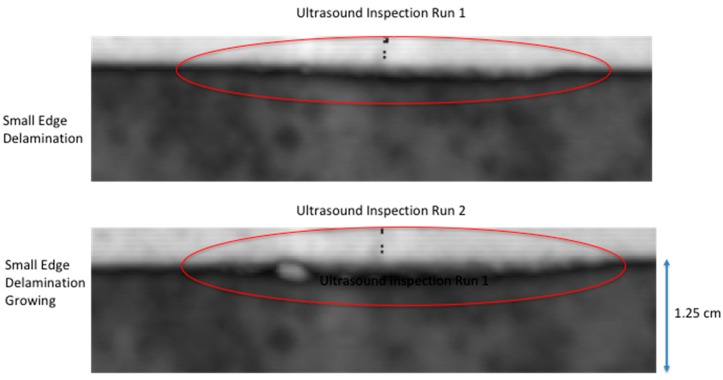
Ultrasonic inspection image after quasi-static second load run with comparison to initial run ultrasonic inspection.

Digital image processing was required to enhance detection of thermal events during load and to facilitate comparison of the thermal inspection imagery to the ultrasonic data. To enhance detection in real time, a delayed subtraction algorithm was implemented [27,28]. Typical parameters of 100 frames were averaged and a delayed subtraction of 20 averaged frames were generally used for real time processing [28]. The delayed subtraction of 20 averaged frames, given a frame rate of 80 Hz (640 × 512 resolution) results in a time delay of approximately 25 s. This was sufficient time to provide contrast of thermal transients that typically last much less than 25 s. The delayed subtraction removed fixed background IR radiation while increasing sensitivity to changes. The thermal damage was identified over camera noise and background reflections using three criteria: observing a bright area that appears suddenly, stays in one location, and then slowly decays in intensity over time. Comparison to ultrasonic data required an image perspective transformation. The image perspective transformation corrected for the IR camera look angle since the optical line of sight was not normal. The image correction is performed by defining 4 points mapped to a new set of 4 desired points (normal view) [29]. Figure 4 shows the typical processed thermal images for an initial quasi-static load. The thermal images show heating resulting from a small edge delamination growing along the edge of the stiffener flange. The thermal damage was identified over camera noise and background reflections using three criteria: observing a bright area that appears suddenly, stays in one location, and then slowly decays in intensity over time. The ultrasonic inspection image is shown in Figure 5. The ultrasonic inspection was performed using a non-immersion inspection system [30]. The ultrasonic data were obtained using a captive water column with a flexible membrane that allows scanning without immersion into a water tank. The ultrasonic transducer used was a 10 MHz broadband transducer, with a 1.27 cm diameter, and 50.8 cm focus. Full waveform capture was performed using a 12-bit digitizer with mechanical scanning resolution of 0.025 cm. The ultrasonic results confirm a successful capture of an early delamination formation using passive thermography. This damage can be grown and characterized during subsequent load runs. The damage grew during the second quasi-static load run as shown in the passive thermography images of Figure 6. Figure 7 shows the corresponding ultrasonic inspection image. The ultrasonic inspection shows the small edge delamination growing slightly inward toward the stiffener hat. The thermal indications, shown in Figure 4 and Figure 6 (circled), prove passive thermography successfully detected the formation and growth of small edge delaminations for this test setup. The thermal indications from the second load run are not as pronounced as the initial load run. This indicates that initial damage formation releases more heat as compared to incremental damage growth. This was observed testing numerous panels. The passive thermography was able to provide the necessary information to detect damage onset and growth.

### 2.5. Detection of Damage from Instantaneous Heating

**Figure 8:**
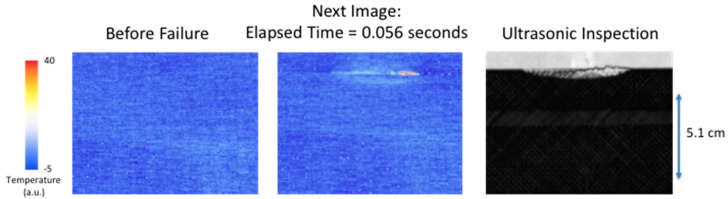
Comparison of instantaneous surface heating to ultrasonic inspection.

**Figure 9:**
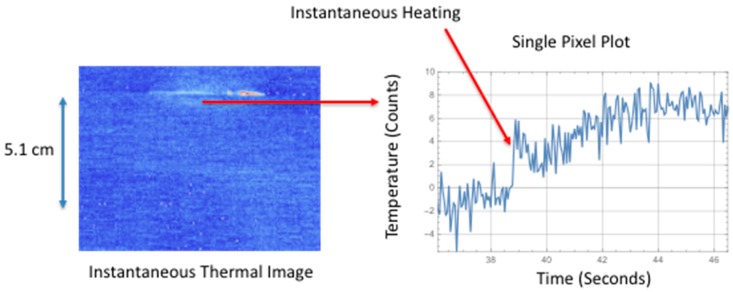
Single pixel plot of instantaneous surface heating.

**Figure 10:**
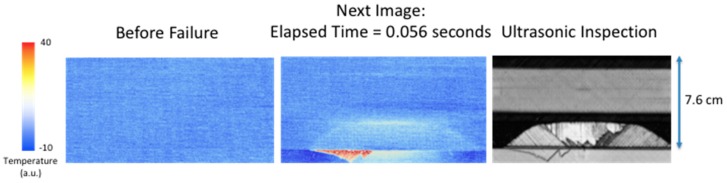
Comparison of large area instantaneous surface heating to ultrasonic inspection.

**Figure 11:**
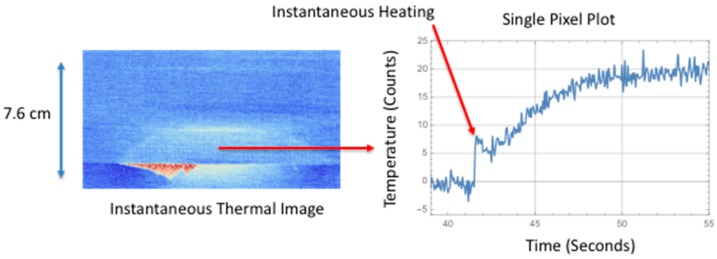
Single pixel plot of instantaneous surface heating for large area failure.

It has been observed the surface temperature, due to larger delamination damage, has two unique components. The first component is an instantaneous rise in temperature and the second component is a slow transient increase in temperature. The first component is most likely due to a combination of factors to include irreversible thermoelastic, plastic deformation, and microstructural heating. The quasi-static loading produces a tension load on the composite that causes deformation. When delamination damage is formed, the tension load is reversed or released, thus causing an instantaneous temperature increase. The second component is due to a mechanical heating at the interface where the damage occurs. This mechanical heating is due to the fracture damage creating friction heating and behaves as an impulse heat source buried at a given depth. This produces a transient temperature rise at the surface. Figure 8, Figure 9, Figure 10 and Figure 11 show the observed instantaneous temperature rise at the surface seen in the example thermal measurements obtained from the seven-point bend tests where the thermal images were acquired at 180 Hz (region of interest of 320 × 320 detectors) and boxcar averaging of 10 frames were applied. A baseline subtraction of an early time image (100 averaged frames producing a time delayed subtraction of 5.55 s) produced the output thermal images. Figure 8 shows the instantaneous thermal response where at 0.056 s the thermally detected damage is similar in size and shape to the ultrasonic inspection. The ultrasonic data revealed the damage, in most cases, formed near the interface between the stiffener flange and skin and grows inward toward the stiffener hat. The damage typically is semi-circular as confirmed by both the thermal and ultrasonic inspection images. The instantaneous heating component can be seen more clearly in Figure 9 where a single pixel, located in the delamination center and halfway down toward hat, is plotted over the damaged region as a function of time.

The instantaneous surface temperature increase occurs at approximately 38.8 s. Afterwards, there is a significant transient increase in temperature at around 40.0 s. This heating is due to the mechanical heating or heat flux generated at the failure interface. This heat diffuses to the surface and the thermal time to reach maximum temperature is a function of damage depth and thermal diffusivity. Similarly, in Figure 10, the instantaneous thermal response for the large area of failure is shown in the middle image where at 0.056 s the thermally detected damage is similar in size and shape to the ultrasonic inspection. The instantaneous heating component can be seen more clearly in Figure 11 where a single pixel, located at the delamination center and halfway up toward hat, is plotted as a function of time. The instantaneous surface temperature increase occurs at approximately 41.7 s. Afterwards there is a dominant transient increase in temperature at around 43 s and this heating is due to the heating at the interface.

Figure 8 and Figure 10 compare the size and shape of the delamination, determined thermally from the instantaneous response to the ultrasonic inspection results. A full width half max calculation, taking into account noise, is used to estimate the crack length of the delamination at two locations. The first location is along the edge of the flange and the second location is half way into the flange toward the hat. These results are compared to the ultrasonic measurements and compiled in Table 2. The thermally estimated crack length is consistently less (to within 25%) than the ultrasonically measured crack length. This is expected since the noise in the thermal signal will affect the length measurements. Another issue is the composite weave pattern introduces surface spatial variations in temperature and thus does not produce a clear boundary. The thermal estimate of the large area damage crack length is in better agreement with the ultrasonic results due to improved thermal signal to noise.

## 3. Theory: Two-Dimensional Simulation for Heating at Damage Interface

**Figure 12:**
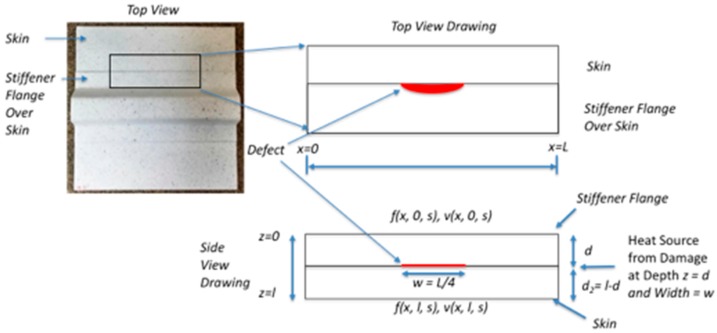
Configuration for two-dimensional model with embedded heat source as a defect.

The transient rise in temperature, as shown in Figure 9 and Figure 11, is due to the fracture damage (mechanical heating) at the interface. This is modeled as an instantaneous heat flux buried at the damage depth. A two-dimensional thermal model is implemented using the quadrupole method presented previously in a paper by Winfree and verified by comparison to finite element modeling [31,32]. The advantages of the quadrupole method are: easily extendable to multiple layers, faster computation time as compared to finite element method simulations, and easy to insert realistically shaped flaws into simulations [31,32]. For this case, shown in Figure 12, with no convection losses at the vertical or horizontal edges, the heat flux generated is at the flaw location depth within the structure. For static loading, the heat flux at the interface buried at a depth d is modeled as an impulse heat flux. A two-layer configuration with top view, top view drawing and side view drawing is shown in Figure 12. The finite lateral dimension, x, is shown in Figure 12 with no heat flow across the vertical edges at *x* = 0 and *x* = L.

The condition of no heat flow across the vertical edges is satisfied if the solution in the *x*-direction is given by a cosine transform. Each of the coefficients of the cosine transform of the two-dimensional Laplace transform temperature solution can be found by dividing the layer in two layers of thickness *d* and *l-d* each with a matrix form given from [31] as:

(1) $$\begin{array}{c} \left[\begin{array}{c} {\tilde{v}}_{m}\left(d,\;s\right)\\ {\tilde{f}}_{m}\left(d,\;s\right)-{\tilde{f}}_{m}^{s}/2 \end{array}\right]\\ =\left[\begin{array}{cc} cosh\left[q_{m}\;d\right] & -\frac{1}{K\;q_{m}}\;sinh\left[q_{m}\;d\right]\\ -K\;q_{m}\;sinh\left[q_{m}\;d\right] & cosh\left[q_{m}\;d\right] \end{array}\right]\left[\begin{array}{c} {\tilde{v}}_{m}\left(0,\;s\right)\\ {\tilde{f}}_{m}\left(0,\;s\right) \end{array}\right], \end{array}$$

(2) $$\left[\begin{array}{c} {\tilde{v}}_{m}\left(l,\;s\right)\\ {\tilde{f}}_{m}\left(l,\;s\right) \end{array}\right]=\left[\begin{array}{cc} cosh\left[q_{m}\;\left(l-d\right)\right] & -\frac{1}{K\;q_{m}}sinh\left[q_{m}\left(l-d\right)\right]\\ -K\;q_{m}sinh\left[q_{m}\;\left(l-d\right)\right] & cosh\left[q_{m}\;\left(l-d\right)\right] \end{array}\right]\left[\begin{array}{c} {\tilde{v}}_{m}\left(d,\;s\right)\\ {\tilde{f}}_{m}\left(d,\;s\right)+{\tilde{f}}_{m}^{s}/2 \end{array}\right]$$

where:

(3) $$q_{m}=\sqrt{\frac{s}{\alpha_{z}}+\frac{\alpha_{x}}{\alpha_{z}}\;{\left(\frac{\pi \;m}{L}\right)}^{2}}$$

and ${\tilde{v}}_{m}\left(z,s\right)$ and ${\tilde{f}}_{m}\left(z,s\right)$ are the cosine coefficients for the temperature and flux respectively and ${\tilde{f}}_{m}^{s}$ is the cosine coefficient for the source flux. The Laplace transforms of the surface temperature and flux are given as ${\tilde{v}}_{m}\left(0,s\right)$ and ${\tilde{f}}_{m}\left(0,s\right)$ respectively. In addition, *K* is the composite thermal conductivity, $\alpha_{z}$ is the thermal diffusivity in the z direction, $\alpha_{x}$ is the lateral thermal diffusivity in the *x* direction, s is the Laplace complex frequency variable, l is the slab thickness, d is the depth of the flaw, m is the Fourier cosine series coefficients for each lateral index of *x*, and L is the slab width. Given damage at depth z = d and no heat flow at the surfaces, Equations (1) and (2) can be solved to find the surface temperature to be as:

(4) $${\tilde{v}}_{m}\left(0,\;s\right)=\frac{{\tilde{f}}_{m}^{s}cosh\left[q_{m}\left(l-d\right)\right]}{K\;q_{m}\;sinh\left[q_{m}\;l\right]}$$

Equation (4) can be solved by taking into account the discrete positions in *x* by the vector representation:

(5) $$V\left(0,s\right)=C\left(s\right).F^{s}\left(s\right)$$

where ***V***(0, *s*) is the Laplace transform surface temperature vector and the elements of the transfer matrix ***C***(s) are given from [32] as:

(6) $$C_{i,\;j}\left(s\right)\;a_{j}\;\frac{\sum_{m=0}^{N-1}a_{m}cos\left(\frac{i\;m\;\pi }{N-1}\right)cos\left(\frac{j\;m\;\pi }{N-1}\right)cosh\left[q_{m}\left(l-d\right)\right]}{\left(2N-2\right)K\;q_{m}\;sinh\left[q_{m}\;l\right]}$$

**Figure 13:**
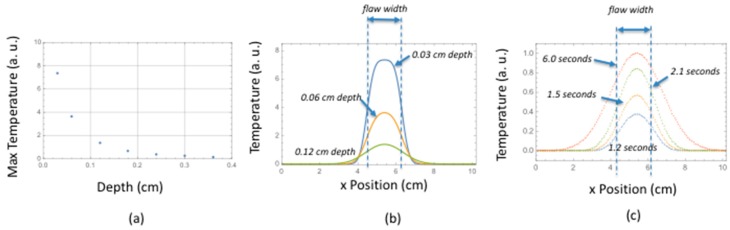
Model results: (**a**) maximum temperature at different source (damage) depths; (**b**) blurring of source edge for different depths; (**c**) blurring of source edge for different times.

Both $a_{j}$ and $a_{m}$ are equal to 1 if the index j (or m) = 0 or N − 1 and 2 otherwise. The Laplace transform source heat flux, $F^{s}\left(s\right)$, is the vector representation of the heat generated during the formation of the flaw along the x dimension at depth z = d and is modeled as an impulse function. For each point, the inverse Laplace transform is calculated at a given time to give temperature as a function of *x* position. The inverse Laplace transform for the surface temperature, V(0,s), is calculated numerically using Talbot’s method [33]. Using the model values given in Table 3, results are shown in Figure 13a–c for maximum temperature as a function of flaw depth, blurring as a function of flaw depth, and blurring as a function of time for a given flaw depth, respectively. The through-the-thickness thermal diffusivity value of the woven layer was determined using a flash through transmission technique and was measured to be $\alpha_{z}$ = 0.0042 cm^2^/s [34]. The lateral thermal diffusivity was estimated to be five times the through-the-thickness value from Dasgupta’s thermal conductivity modeling predictions for woven composites where the lateral thermal conductivity can be a factor of four to six times the through-the-thickness conductivity [35]. This value was set to $\alpha_{x}$ = 0.021 cm^2^/s. The woven composite thermal conductivity is estimated to be *K* = 0.0045 W/cm/K [36]. As can be seen from Figure 13a (left plot), there is a considerable temperature drop for sources that are deeper. This is especially problematic if one takes into account camera noise. Additionally, for deep sources, there is considerable blurring of the edges of the source at maximum temperature. This is shown in Figure 13b plot for sources buried at 0.03, 0.06 and 0.12 cm for maximum temperature time values of 0.16, 0.40, and 1.04 s, respectively. There is also considerable blurring over time as shown in Figure 13c for a source buried at 0.24 cm.

## 4. Results: Comparison of Simulation to Measured Thermal Response

**Figure 14:**
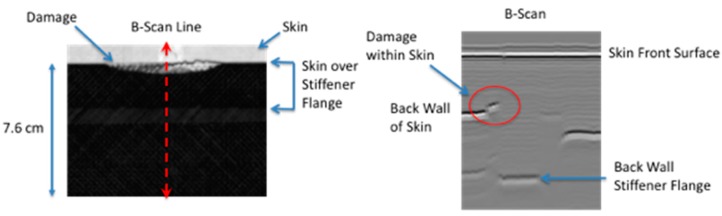
Ultrasonic data to estimate damage depth for small area damage.

**Figure 15:**
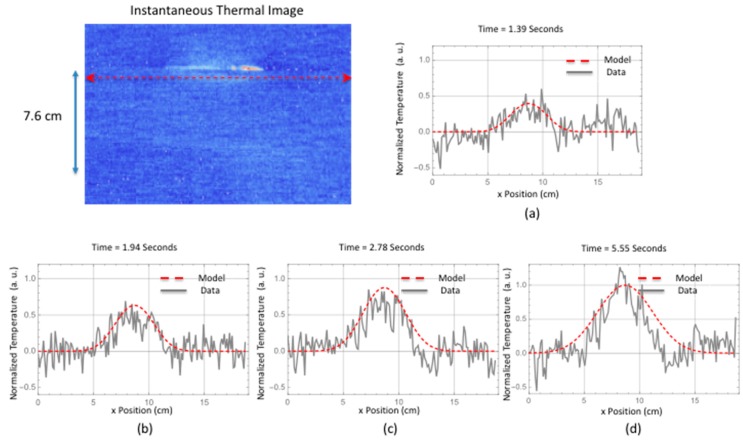
Two-dimensional simulation comparison to surface temperature response as a function of time for a flaw source buried at 0.258 cm.

**Figure 16:**
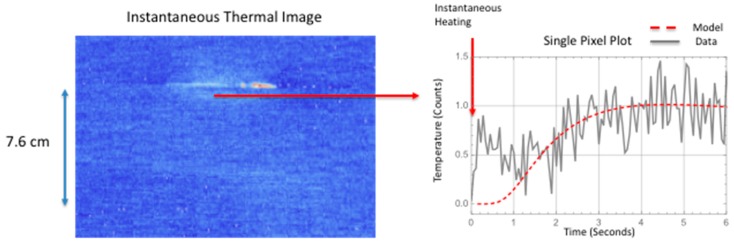
Temporal pixel plot comparison to model response for flaw source buried at 0.258 cm.

A comparison is made between the two-dimensional simulation and the measured thermal response for a delamination located near the interface between the stiffener flange and skin. The instantaneous thermal response provides an estimate of the damage size and start time. The damage depth was estimated using the ultrasonic data. This is shown in Figure 14 where the ultrasonic B-scan inspection of the small area failure reveals damage within the skin. The skin is 12 plies thick. Since the thermal data were obtained from the hat side, the depth of damage would be the stiffener flange thickness (12 plies with a thickness of each ply @ 0.020 cm each for a total thickness of 0.240 cm) plus one skin ply (0.018 cm thick) for a total damage depth 0.258 cm. The thermally estimated damage width, from Table 2 for a line within the delamination located halfway down toward hat, was used in the simulation. Shown in Figure 15 is the comparison of the two-dimensional thermal simulation to the surface temperature response as a function of time, for a flaw source buried at 0.258 cm. By estimating the noise level, the thermal responses were offset corrected and normalized to the maximum temperature obtained at 5.55 s. The simulation response was also normalized at the same time. Figure 15c,d show the simulation slightly overestimates the lateral size (right side of plots) as compared to the thermal data. This perhaps is due to an overestimation of the lateral diffusion or variation in the delamination crack depth due to both intralaminar or interlaminar damage. Shown in Figure 16 is a temporal single pixel plot comparison to the simulation response for flaw source buried at 0.258 cm. The start time is determined from the instantaneous response. As can be seen, the early time is dominated by the instantaneous heating.

**Figure 17:**
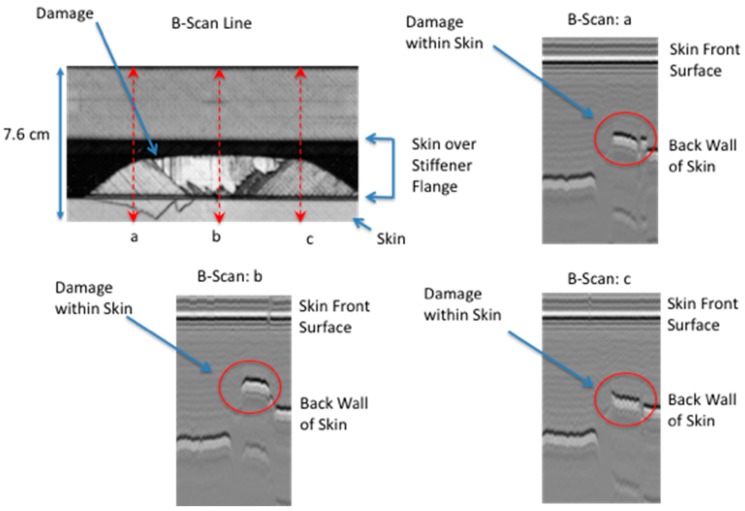
Ultrasonic data to estimate damage depth: (**a**) and (**c**) indicate damage one ply within skin; (**b**) indicate deeper damage two plies within skin.

Shown in Figure 17 is the ultrasonic B-scan inspections for the large area failure where the damage is at multiple interfaces. The B-scan images reveals estimated damage depth within the skin to be from one ply deep (B-Scan: a and c) and two plies deep (B-Scan: b). The skin is 12 plies thick. The thermally determined damage width, from Table 2 for a line within the delamination located halfway up toward hat, was used in the simulation.

**Figure 18:**
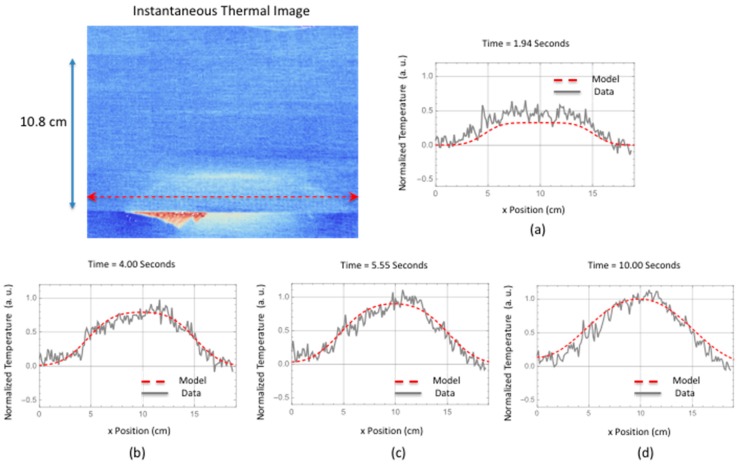
Two-dimensional simulation comparison to surface temperature response as a function of time for a flaw source buried at 0.276 cm.

**Figure 19:**
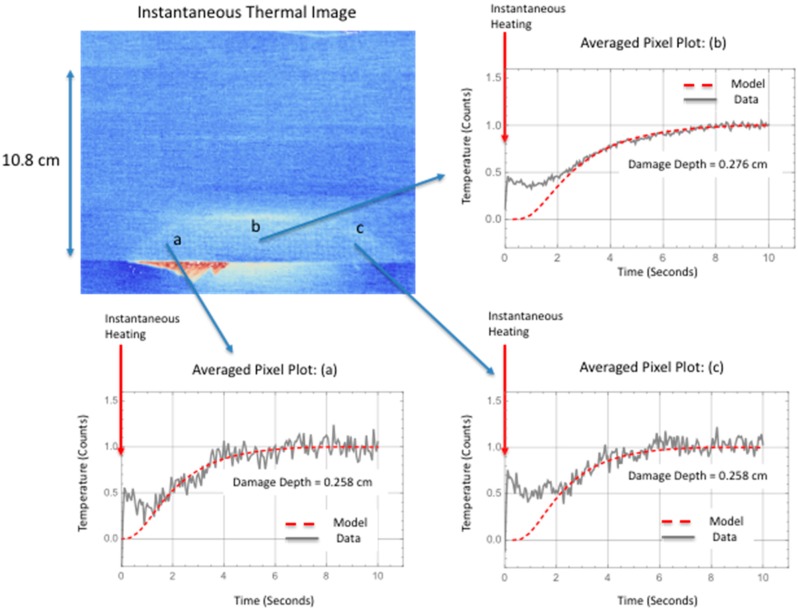
Temporal pixel plot comparison to model response for large area delamination at various locations.

Shown in Figure 18 is the comparison of the two-dimensional simulation to the surface temperature response as a function of time, for a flaw source buried two plies within the skin. The stiffener flange is 0.24 cm plus two skin plies (0.018 cm thick each) which gives an averaged damage depth of 0.276 cm. Estimating the noise level, the thermal response was offset corrected and normalized to the maximum temperature obtained at 10.00 s. Fairly good agreement between the thermal simulation and the data is obtained for all the plots except the early time plot Figure 18a. The early time plot error is due to the instantaneous heating which is more pronounced for the large area failure at the early times. Shown in Figure 18d, the simulation slightly overestimates the lateral size but is in better agreement as compared to the small delamination shown in Figure 15d. The better agreement in Figure 18d is most likely due to an overestimation of the lateral diffusion in combination with the left and right delamination depth being more shallow. Shown in Figure 19 are temporal plot comparisons to the model response at different locations on the large area damage. As can be seen, the early time is dominated by the instantaneous heating, similar to the temporal plot in Figure 16, for all three averaged pixel plots. The plots were generated by averaging a 5 × 5 pixel area. The fitting response is shown in Table 4 where the mean squared difference between the model response and the thermal data is calculated for different thicknesses. The mean squared difference is calculated for a time window of 1 to 10 s to remove the influence of the instantaneous heating that is not included in the model. The results in Table 4 show, by minimization of the mean squared error, the middle area b damage is at a depth of 0.276 cm and the left and right areas a and c respectively is at a depth of 0.258 cm. This is in agreement with the B-scan results in Figure 17 that show the delamination is buried deeper for middle area B-scan line (b) as compared to B-scan lines (a) and (c) which indicate the damage is less within the skin layer and therefore not as deep.

## 5. Discussion

There have been numerous studies using thermography to detect damage in a composite structure during load testing. The early work by Reifsnider and Henneke involved damage detection using cyclic or fatigue loading [14,15]. Subsequent thermography efforts have mainly focused on fatigue loading for passive thermography [16,17,18,19,37,38]. Previous works of passive thermography applied during static loading were mainly focused on coupon level testing [23,24,25,26,27]. We have demonstrated passive thermography as a tool to detect damage in a hat-stiffened composite panel in real time. This has been possible with newer more sensitive IR cameras, wide bandwidth digital camera interfaces via GigE, and faster computers allowing for real time processing, storage, and display of processed imagery. Passive thermography was used to successfully detect the initiation of small edge delaminations in a composite hat-stiffened panel and control its growth during quasi-static load testing. This was shown in Figure 4 through Figure 7. By precisely controlling the damage growth, documentation of the damage progression modes can be made using a more detailed inspection method such as ultrasound or X-ray CT.

In addition, it has been observed that the temperature rise due to damage formation at the surface is composed of two different heat generation mechanisms. The first is instantaneous and conforms to the shape of the damage. This instantaneous release of heat over the damaged area is due to the strain release when damage is formed. This instantaneous response was observed to provide an estimate of overall damage size as compared to the ultrasonic results. This is shown in Figure 8 and Figure 10. The second response is the transient increase in temperature due to mechanical heating at the interface during failure. This is clearly seen in pixel plots of Figure 16 and Figure 19 for both small and large delaminations. By using the ultrasonic data for damage depth estimation and the instantaneous response for the thermal start time and to estimate damage size, a two-dimensional multi-layered thermal simulation was compared with the thermal data as shown in Figure 15 and Figure 18. For Figure 15d and Figure 18d, the model slightly overestimates the crack length. This could be due to an overestimation of the lateral diffusivity value. This indicates the importance of an independent measurement of the in-plane thermal diffusivity for defect sizing. Shown in Figure 19 are the thermal data and simulation for several temporal plots over the large area damage. The thermally measured damage depth was confirmed from the ultrasonic B-scan results shown in Figure 17. This indicates that the thermal model could be used to determine damage depth at certain averaged pixel locations. A limitation of the 2-Dimensional model is to define the delamination damage at various depths. The 2-Dimensional model would need to be modified to add more layers with heat sources at different depths. Lastly, future work would also require modifying the thermal model to take into account the instantaneous heating. This would allow for early time model fitting.

## 6. Conclusions

Passive thermography was used to successfully detect the initiation of small edge delaminations in a composite hat-stiffened panel during quasi-static seven-point load testing. By limiting the damage growth to small increments, documentation of the damage progression modes can be made using a more detailed inspection method such as ultrasound and X-ray CT. In addition, a method is presented where the damage depth could also be determined by taking into account the instantaneous response as the thermal start time and using the model to temporally fit the data. Real-time measurement of the crack depth, revealing damage growth migration to a different interface, would be of great value during structural load testing.

## Figures and Tables

**Table 1:** Specifications of the IR camera.

IR Camera	Specifications
Detectable Wavelength	3–5 microns
Detector Type	InSb
Total Number of Detectors	640 × 512
Temperature Resolution	<18 milli-Kelvin
Pixel Pitch	15 × 15 microns
Frame Rate	80 to 180 Hz
Dynamic Range	14 bits

**Table 2:** Comparison of Thermal to Ultrasound Measurement of Crack Length.

Delamination And Location	Thermal Estimation of Crack Length (cm)	Ultrasonically Measured Crack Length (cm)
Small Delamination along Edge	4.8	6.2 ± 0.05
Small Delamination Halfway down toward Hat	3.5	4.7 ± 0.05
Large Delamination along Edge	14.1	15.0 ± 0.05
Large Delamination Halfway up toward Hat	10.6	12.1 ± 0.05

**Table 3:** Model values.

Model Variable	Value
*l* (thickness of flange + skin)	0.46 cm
Instantaneous Heat Flux	1.0 Watt/cm^2^
Flaw Position	4.3 to 6.3 cm
*L* (Slab Width)	10.2 cm
$\alpha_{z}$ (through thickness thermal diffusivity)	0.0042 cm^2^/s [25]
$\alpha_{x}$ (lateral thermal diffusivity)	0.021 cm^2^/s [26]
*K* (thermal conductivity)	0.0045 W/cm/K [27]
*N (Discrete positions in x)*	160

**Table 4:** Temporal plot comparison of model to the thermal data.

Thickness (cm)	Pixel Plot (a) Mean Squared Difference	Pixel Plot (b) Mean Squared Difference	Pixel Plot (c) Mean Squared Difference
0.240	0.0165	0.0139	0.0150
0.258	0.00965	0.00578	0.0105
0.276	0.0100	0.00326	0.0139
0.294	0.0188	0.00755	0.0251
0.312	0.0338	0.0172	0.0419

## Nomenclature

$\alpha_{x}$	=	Lateral thermal diffusivity
$\alpha_{z}$	=	Through-the-thickness thermal diffusivity
***C***	=	Transform matrix
*d*	=	Depth of defect
*i*	=	Transform matrix iterator
$\tilde{f}$	=	Laplace transform of heat flux
$F^{s}$	=	Laplace transform of source heat flux (vector representation)
*j*	=	Transform matrix iterator
*K*	=	Thermal conductivity of woven composite
*l*	=	Overall thickness of flange + skin
*L*	=	Slab width
*m*	=	Cosine series coefficient index
*N*	=	Number of evenly spaced locations along x
*s*	=	Laplace complex frequency term
***V***	=	Laplace transform of surface temperature vector (vector representation)
$\tilde{v}$	=	Laplace transform of temperature
*x*	=	Lateral dimension
*z*	=	Depth dimension
