# Erratum: Zalameda, J. et al. Detection and Characterization of Damage in Quasi-Static Loaded Composite Structures using Passive Thermography. *Sensors* 2018, *18*, 3562

**DOI:** 10.3390/s18113977

**Published:** 2018-11-15

**Authors:** Joseph Zalameda, William Winfree

**Affiliations:** NASA Langley Research Center, MS231 Hampton, VA 23681, USA; william.p.winfree@nasa.gov

The authors wish to correct Table 3 in their paper published in *Sensors* [1], doi:10.3390/s18103562, https://www.mdpi.com/1424-8220/18/10/3562. The following corrected table should be used with incorrect references removed from original. The corrected table does not change the scientific results. The correct references are used in the original manuscript. The manuscript will be updated and the original will remain online on the article web page, with a reference to this Erratum.

## Figures and Tables

**Table 3:** Model values.

Model Variable	Value
*l* (thickness of flange + skin)	0.46 cm
Instantaneous Heat Flux	1.0 Watt/cm^2^
Flaw Position	4.3 to 6.3 cm
*L* (Slab Width)	10.2 cm
$\alpha_{z}$ (through thickness thermal diffusivity)	0.0042 cm^2^/s
$\alpha_{x}$ (lateral thermal diffusivity)	0.021 cm^2^/s
*K* (thermal conductivity)	0.0045 W/cm/K
*N (Discrete positions in x)*	160
